# Potentials for Improving Support and Care to Survivors of Sexual Violence – a Case Study Within a Multiorganizational Setting in Sweden

**DOI:** 10.5334/ijic.8995

**Published:** 2025-09-09

**Authors:** Helena Kilander, Caroline Lyssarides, Cecilia Fredlund, Bertil Lindenfalk, Sofi Fristedt, Annika Nordin

**Affiliations:** 1 Jönköping Academy of Improvement of Health and Welfare, School of Health and Welfare, Jönköping University, Jonkoping, Sweden; 2 Department of Women’s and Children’s Health, Karolinska Institutet, and the WHO collaborating centre, Karolinska University Hospital, Stockholm, Sweden; 3 Department of Gynaecology and Obstetrics Eksjo, Region Jönköping County, Sweden; 4 Department of Psychiatry in Linköping, and Barnafrid –the Swedish national knowledge center concerning violence against children Department of Biomedical and Clinical Sciences, Linköping University, Linköping, Sweden; 5 Department of Caring Sciences, Faculty of Medicine, Lund University, Sweden

**Keywords:** co-production, integrated care, sexual abuse, quality improvement, victims

## Abstract

**Introduction:**

Worldwide, exposure to sexual violence has widespread consequences for survivors’ health and for their communities. There is still insufficient understanding of how to design support and care services for survivors in a multiorganizational context. This study aims to map and explore support and care for survivors of sexual violence from a multiorganizational perspective in Sweden, to identify and understand common improvement areas.

**Method:**

This study was conducted within a larger project called *Survive* which aims to improve support and care for survivors of sexual violence in Jönköping County in Sweden. This case study integrates qualitative data from interviews (n = 34) and an electronic survey (n = 95) with participants from eight organizations that provide support and care to survivors of sexual violence. Qualitative data was analysed using thematic analysis. The quantitative data from the electronic survey was evaluated to map the ‘organizations’ involvement in support and care provision by using descriptive statistics.

**Results:**

The findings encompass four themes to improve support and care: 1) Systematic approaches to identifying exposure to sexual violence; 2) A need to improve the integration of existing supportive resources; 3) Need for an interlinked process for trauma therapy and long-term support; 4) The potential of developing capacity, competence and teamwork in trauma care.

**Conclusion:**

It is essential to improve support and care for survivors of sexual violence by developing systematic approaches for identification of sexual violence, interlinked support and care processes as well as improved access and competence in trauma care.

## Introduction

Globally, sexual violence is a public health issue with far-reaching consequences for survivors’ health, future relationships and society [1,2]. The United Nations Sustainable Development Goals call for elimination of violence against all women and girls in the public and private sphere, including trafficking and sexual exploitation (2, target 5.2).

This study adopts the World Health Organization’s definition of sexual violence as “any sexual act, attempt to obtain a sexual act, unwanted sexual comments or advances, or acts to traffic or otherwise directed against a person’s sexuality using coercion, by any person regardless of their relationship to the victim, in any setting, including but not limited to home and work” [3].

In Sweden, self-reported subjection to sexual violence has increased since year 2013 [4]. It is possible that the #MeToo movement and changed legislation have resulted in both increased identification and report of sexual violence [5]. In a Swedish study including 3282 responses in 2022, one in four students in secondary school, regardless of sex, reported experiences of sexual violence before the age of 18 [6]. Similar trends are reported in a Swedish study including a population-based sample where approximately 22 % of the men and 26 % of the women disclosed experiences of sexual violence in childhood [7].

Compared to the general population, survivors of sexual violence more commonly experience psychiatric diagnoses [8] and other medical problems [9], and their need of access to health care services is in many cases greater [10,11]. Sexual violence is one of the most common reasons for developing symptoms of post-traumatic stress disorder (PTSD) globally [12]. The absence of a physical cause for symptoms of PTSD among help-seeking survivors [13] poses a challenge to existing systems of support and care, as previous experience of sexual violence as a cause is seldom explored in clinical settings. Taken together, this reflects the need for improved interprofessional, interorganizational collaboration between different providers regardless of the age of survivors. However, research on how to improve such collaboration and what it should include is scarce.

Helping survivors disclose their experiences of sexual violence and offering integrated, person-centred, organized support and care services could ultimately improve their health [1,14]. Rarely, however, do survivors disclose their experience of sexual violence spontaneously [11,15]. Still, there are barriers in providing support and care for survivors of sexual violence in high-income countries such as Canada, the United States and Sweden [11,16,17,18]. Common barriers to disclosing exposure to violence could be lack of communication, the absence of a non-judgemental approach in meetings with actors in different settings, competing priorities, lack of available resources, and missing links between resources, services and referrals to facilitate physical access to services for survivors [11,16,17]. While the need for a comprehensive model of integrated care and support within multiorganizational settings – including the legal system – has been suggested, most existing studies concerning barriers to care and support focus on individual institutions such as individual hospitals, primary care units or specialized care such as sexual assault services [16,17,18]. Previous studies are conducted in contexts where specialised care are provided [11,16,17,18]. Few studies explore rural and multiorganizational settings engaging both the public sector and civil society and all age groups in this field.

A Swedish report pointed to several improvement areas in the care for survivors of sexual violence, including ensuring a more knowledge-based, equal and resource-efficient care [19]. Emergency and follow-up care related to sexual violence is provided in different and unequal ways in the different geographical health care regions across Sweden. In some Swedish counties there are specialized centres for providing care related to sexual violence, yet in others, care is provided within the regular health care system. For women, this care is mainly provided at hospital departments of obstetrics and gynaecology [19]. During investigations and court proceedings, many health care regions also provide integrated services for children exposed to violence, where different providers work together to ensure that victimized children and their families receive emergency social support care, trauma support and healthcare [20]. However, guidelines for emergency health care services regarding children, men, transgender and non-binary groups at the time of sexual violence are missing [19,21].

In Sweden, many organizations are involved in the support and care of survivors of sexual violence [19]. In this study, health care services, social services, school and student health services, integrated services for children, the police and non-governmental organizations (NGOs) are identified as organizations that provide low-threshold support and care for child and adult survivors of sexual violence in Sweden. NGOs such as women’s shelters have historically played an important role in providing support for survivors [19].

There is a growing body of knowledge and promising evidence of how participatory research and co-production methodologies are being employed to address health disparities and service redesign and improve services among socioeconomically disadvantaged populations [22,23,24]. Less is known about how such support and care for survivors regardless of age should be co-produced, integrated and coordinated within a multiorganizational setting targeting survivors of sexual violence where no specialized centre is available.

## Aim of the study

This study aims to map and explore support and care for survivors of sexual violence regardless of age from a multiorganizational perspective in Sweden, to identify and understand common improvement areas.

## Research Methods

### Involvement of people with lived experience

This study was conducted within a larger project called *Survive*, aimed to identify areas for improvement in the current care and support system to design and test proposals for interventions, regardless of relationship and gender identity [25]. In this project, Participatory Action Research was chosen as the overall theoretical framework, where the design work was based on the Experienced based co-design (EBCD) [26]. Due to the risk of re-traumatizing survivors of sexual violence the Survive project design does not directly include people with lived experience. Instead, collaboration with organizations representing, and working for, these individuals were included. Furthermore, the use of Personas facilitated the ongoing focus on the experiences of people with lived experiences.

Two NGOs representing people with lived experiences of sexual violence were partners in this project. Four representatives from these organizations contributed to different parts of the research project, such as the design of the questions in the electronic survey, the parts of the project involving co-design, and the analysis of the overall results in the project from a user perspective, including writing a project report in Swedish.

### Setting

The study took place in Jönköping County, a mixed rural and urban county in the southern part of Sweden that includes 13 municipalities, two district hospitals and one county hospital, providing health and welfare services for about 360 000 citizens [27]. There is no specialized centre available for providing care related to sexual violence, so it is provided within the regular health care system.

### Design

**Figure 1:**
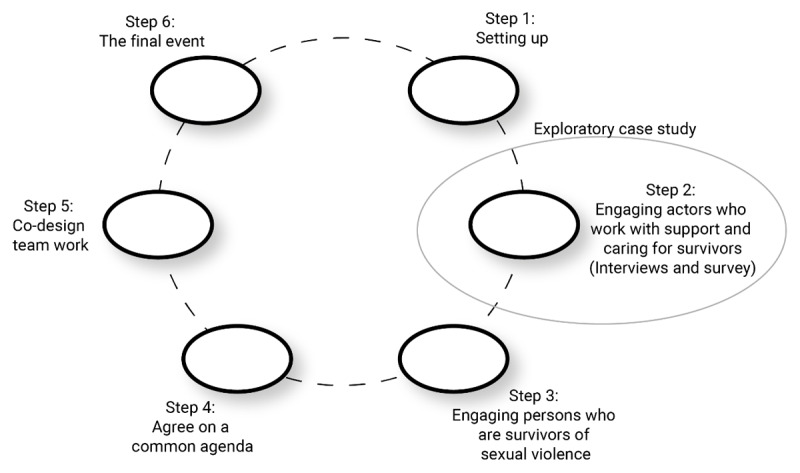
Description of the EBCD process.

Using an exploratory case study design [28], we combined data from interviews and an electronic survey collected in the EBCD process (step 2, Figure 1) to meet the study aim. Case studies are a suitable research approach when seeking to generate an in-depth, multi-faceted understanding of a complex issue in its real-life context [28]. The qualitative data set included transcripts from individual interviews, joint interviews, and focus group discussions with actors involved in support and care of survivors of sexual violence. In addition, an electronic survey including both closed and open-ended questions was conducted to map the organizations engagement. The methods are reported in accordance with the Consolidated Criteria for Reporting Qualitative Research (COREQ) checklist [29].

### Sample and procedure

Purposive sampling [30] was used to capture different perspectives and rich experiences from the field. Experts including two NGOs, researchers, the project leader and clinicians with experiences in the field were invited to several co-design meetings to jointly identify potential organizations of relevance for the study, based on previous reports [19].

A wide variety of actors from the health care services were invited, including departments of obstetrics and gynaecology, psychiatric clinics, primary care units, adolescent clinics and integrated services for citizens including children. In addition, social services, the local police authority, school and student health services, and NGOs in the geographical area were invited to participate in the study. Information about the study purpose and procedure was given by email or orally at staff meetings between September 2022 and May 2023.

Initially, 62 individuals showed interest in participating in the focus group discussions or interviews. Ultimately, 34 gave their consent to participate, of which 30 were women and four were men. It was up to the participants to choose the interview format they found the most convenient. Of the 34, 20 were recruited by email or orally at staff meetings in the first step, and in the second step another 14 were recruited by using a snowball sampling strategy.

Ninety-five individuals answered the closed questions in the electronic survey and these data were included in the quantitative analysis. Eighty individuals also completed the open-ended questions in the survey and these data we included in the qualitative analysis. The electronic survey was open during two months in spring 2023 and reminders were sent via email twice. The quantitative data was evaluated to map the ‘organizations’ involvement in providing support and care of survivors of sexual violence by using Excel software and is presented as descriptive data in the result section.

An interview guide was developed by the authors [HK, SF, AN and CF], and feedback was given by representatives from two NGOs and two actors who did not participate in the study [supplementary 1]. The electronic survey included four closed and two open-ended questions as well as follow-up questions [supplementary 2] and was developed by representatives from interest groups and two of the authors of this study [BL and HK]. Actors involved in the support and care of survivors of sexual violence are referred to as participants in the findings and discussion section of this paper.

Before starting the focus group discussions, interviews or joint interviews, participants received information about the study once more and gave their written informed consent. Six focus group, three joint interviews and two individual interviews were conducted. The focus group discussions had three to six participants, and the joint interviews had two participants. Three authors [HK, CL or AN] took turns participating as moderators. Seven focus group discussions and joint interviews took place in meeting rooms at the University, the health care services or the local police authority. Additionally, two focus groups and two interviews were performed digitally and lasted for a median length of 67 minutes [ranging from 44 to 84 minutes]. All interviews were digitally recorded and transcribed verbatim. The transcribed data from focus group discussions, interviews and open-ended answers in the digital survey contained 165 pages of text.

### Analysis

A reflexive thematic analysis was conducted and followed six phases according to Braun and Clarke [31]. In the first step, the first author [HK] became familiar with the data by reading through the material and noting patterns. In the next step, meaning units relating to the study aim were identified and initial codes were created and arranged by HK using NVivo version 1.7. The codes were re-read and potential themes were generated. Then, the themes were revised by reading the collated citations for each theme. The analysis concerned seeking connections between codes and themes in collaboration between HK and CL. In accordance with Braun and Clarke, the analysis moved back and forth through the steps of the process. HK and CL created a map of themes including all material. The first author, HK, shared preliminary themes with most of the interview participants at a workshop before the final revision of the main themes. While two of the authors [HK and CL] had the main responsibility for the process of analysis, the final themes were discussed and agreed upon by three of the authors [HK, CL, AN].

### Reflexivity

The study design has enabled reflexivity throughout the project due to close collaboration with actors and interest groups representing people with lived experiences of sexual violence. Additionally, three of the authors [HK, CL and CF] have clinical experience of providing care and support for individuals who have been exposed to sexual violence. Their preunderstandings were used when designing the study and interpreting data. Four of the researchers [AN, HK, BL and SF] also have extensive experience of co-design and improvement work in health and welfare services.

### Ethical considerations

This study was approved by the Swedish Ethical Review Authority [Dnr 2022-02146-01]. The research group reflected on potential risks for re-trauma regarding sharing survivors’ perspectives about support and care in the overall project *Survive*. Due to these risks, NGOs were engaged to represent people with lived experiences of sexual violence, rather than engaging survivors in the co-design process.

## Results

An overview regarding the eight organizations represented in the qualitative data collection is presented in Tables 1 and 2. According to the electronic survey, more than half of the support reported in the survey [60%] was offered by the health care services, and most of the provided support [58%] targeted adults rather than younger persons. The need to organize and use existing resources of both emergency and long-term support more efficiently was evident. A total of 83% of the participants in the survey reported that they offered support in the context of sexual violence. Regarding the distribution of this support, 27% reported emergency social care support, 45% long-term support and 11% provided both emergency social care support and long-term support.

**Table 1:** Organizations represented in the focus group discussions or interviews [n = 34].


TYPE OF ORGANIZATION	NO. OF PARTICIPANTS

Social services	7

School and student health services	3

Health care services	19

Police	3

Integrated services for citizens including children*	2


Gender distribution: Women = 30, Men = 4. *Collaboration between health care, social services and police.

**Table 2:** Distribution of organizations represented in the electronic survey [n = 95].


TYPE OF ORGANIZATION	RESPONDENTS IN NUMBERS

Health care services	57

Social services	24

NGOs*	5

Private health care centres	5

Integrated services for citizens including children**	4


*NGOs = Non governmental organisations include; women’s shelter, victim support organizations and churches proving help and support for survivors free of charge.**Collaboration between health care and social services.

The thematic analysis uncovered four main themes (Table 3): 1) Systematic approaches to identifying exposure to sexual violence, 2) A need to improve the integration of existing supportive resources, 3) Need for an interlinked process for trauma therapy and long-term support and 4) The potential of developing capacity, competence and teamwork in trauma care. The four main themes resulted in one overarching theme: “Perspectives on developing seamless pathways and coordination of support and care in a multiorganizational setting”.

**Table 3:** Overarching theme, main themes and sub-themes in the final analysis.


OVERARCHING THEME	MAIN THEMES	SUB-THEMES

Perspectives on developing seamless pathways and coordination of support and care in a multiorganizational setting	Systematic approaches to identifying exposure to sexual violence	Screening of risk groups vary

		Systematic approaches can facilitate identification of victims

		Needs of routines despite of gender

		Individual factors among provider s affect trust and identification of violence

	A need to improve the integration of existing supportive resources	Different actors with unclear roles and missions

		Underutilised recourses for emergency social care support

		Support after sexual violence is limited by gender and relationship status

		Needs of coordinating emergency and long-term support

		Different ways of entering the organizations for support

	Need for an interlinked process for trauma therapy and long-term support	Needs of interlinked support and trauma care processes

		A call for united process to establish trust among survivors

		Challenging consultations with survivors with co-morbidities

		Barriers in long-term support and trauma therapy

		Needs of life long-lasting support outside existing health care systems

		Limited support after exposure to sexual violence online

	The potential of developing capacity, competence and teamwork in trauma care	Willingness to improve encounters of survivors

		Feelings of frustration due to organisational capacity

		Limited knowledge in trauma care in the context of sexual violence

		The value of teamwork and shared learning

		Needs of support and recovery for professionals involved in support and care for survivors


Seamless pathways refer to the need of developing links between the identification of exposure to sexual violence and resources and referrals to services for survivors of sexual violence. Present pathways and coordination of support and care were different for survivors of sexual violence depending on age. For children, seamless pathways and coordination of support and care existed within integrated services for children who were exposed to violence, but only during a police investigation. For adults, seamless support and care was limited to women within programmes for preventing intimate violence.

### Systematic approaches to identifying exposure to sexual violence

Systematic approaches to identifying exposure to sexual violence among survivors varied across the different organizations. Participants described that the use of systematic approaches such as structured questions often depended on personal approaches to care provision and could thus be improved. For an example structured questions were applied when screening for exposure to intimate violence during pregnancy in maternal health care. Furthermore, screening for exposure to sexual violence was reported in neurodevelopmental investigations in the psychiatric care. However, participants representing social services reported a lack of systematic approaches targeting exposure to sexual violence among children and young people living in known adverse circumstances.

“When it comes to children and young people [in social services], I think that perhaps we should have introduced … a battery of questions about sexual violence and sex for compensation … young people should be asked in all cases.”

“I agree that it is a shortcoming that we do not ask systematically. We do already know that Care of Young Persons within [the Swedish Care of Young Persons Act, SFS 1990:52] [32] involves a higher risk for all types of victimization of violence including sexual violence … We still often fail in asking.”

– Participants, Social services, joint interview

Participants stressed that both organizational and individual factors influenced whether they brought up exposure to sexual violence. Individual factors were described as, for example, limited knowledge on how sexual violence could be manifested and defined. Additionally, varying ability to establish trust in meetings with survivors and how to initiate questions about violence were mentioned. Participants across different organizations reported struggles in how to identify, define and document previous or current exposure to sexual violence. Organizational factors such as lack of time, lack of systematic approaches, lack of guidelines, low prioritization and limited trust in the organizational capacity to deal with the assessment of needs of support and trauma care were identified.

“I don’t think there is any action plan at all for this target group. Would be good with guidelines on how we in school should ask [and] act; [it happens] easily that we do too much and maybe eliminate evidence if the abuse takes place in children of school age.”

*–* Participant 5, School health services, electronic survey

### A need to improve the integration of existing supportive resources

Participants across organizations reported being involved in providing emergency social care support to survivors in hospitals or social services. In general, participants reported few experiences of providing emergency social care support for men and non-binary or transgender adults.

Participants from the social and health care services and within the local police reported unclear roles regarding which organizations should be available or involved in providing this support and to what extent. In emergency services at hospitals, the support for rape victims among female adults and children was often described as functioning well. For female adults, emergency social care support was often described as being offered within the first month.

For children, this support was often described during the initial phase of investigation and court proceedings within the integrated services for children exposed to violence. However, participants described that the coordination of emergency social care support for children needed to be improved since integrated services only covered needs during a police investigation.

“Emergency social care support after being raped works well for female adult survivors within the health care system. … you try to prioritize these particular cases as well … to be caught as quickly as possible, so I feel that accessibility works.”

“I agree with you … I also think that there is a good availability and opportunities to be able to catch more survivors in the near future than we reach today.”

– Participants, Health care services, Focus group 3

Participants representing both social and health care services reflected on underutilized resources for emergency social care support after sexual trauma within their own organization, as well as in relation to other organizations. They experienced that they failed in reaching out as they were seldom contacted by survivors or the police in cases of legal processes. They reflected on many organizations being involved and doubted whether other organizations were aware of them as available resources.

Taken together, participants across organizations stressed the need of coordinating existing resources of support more efficiently. They also called for making emergency social care support more visible within their own organization and accessible to survivors of sexual violence regardless of gender or relationship status.

“I’m not sure if they know that the social emergency services exist and are available, … that there is another availability for emergency social care support than just office hours.”

– Participant K, Social services, interview

“We have a very large and competent network that we turn to when the need of support after sexual trauma arises. We can definitely initiate provision of emergency social care support for the victim to a larger extent …”

– Participant 51, Interest group, electronic survey

### Need for an interlinked process for trauma therapy and long-term support

Participants across the organizations emphasized the need for interlinked processes for coordinating long-term support and trauma care to establish trust among survivors. Such a interlinked process was envisioned as a unified process for all survivors, regardless of legal processes, age or gender, like the structure of care within integrated services for victimized children. Participants across the organizations highlighted barriers in providing long-term support and trauma therapy for survivors with PTSD. Barriers mentioned were unclear processes and limited physical access to trauma therapy. Participants described how these challenges affected trust and survivors’ willingness to seek help.

The desired interlinked process included criteria for access to trauma therapy, care flows, links between resources and referrals for support and trauma care as well as clearer roles and responsibilities for providing long-term support and care. Participants stated that the region health care and the municipals in the area already collaborated and facilitated coordination of support in mental health problems affecting children in general. They referred to the service called “one way into the system”, where all support and care for children with mental health problems was available and coordinated from the same platform.

Similar interlinked processes were available for children within legal processes after exposure to violence, through the integrated services for children, but not for adults. Participants stressed that different organizations such as health care services, social services and the police should learn from existing services for children with mental health problems and offer an interlinked process including trauma care and long-term support for adults as well:

“We see a need for the support and care process to be more explicit in who should work with the target group. In the social services, we get cases that might get better care, but they don’t always want to receive that support from health care services … There is a risk that they will be bounced around and not have access to the support that is available … Some young people feel that the situation got worse after they reported a sexual crime, that it tore open the wound again … and that they were left in it …”

– Participant 25, Social services, electronic survey

Participants representing health care services, social services, the police and interest groups described challenging consultations with survivors of sexual violence in need of interlinked, individualised and trustful care processes. This need was especially highlighted in cases with survivors facing co-morbidity, who often had lost trust in receiving help or were hard to reach. Co-morbidity was often defined among survivors who, besides sexual violence, had substance abuse problems, sold sexual services, or suffered from a neuropsychiatric diagnosis or intellectual disabilities. Participants described that it took time to establish trust among those who also were facing co-morbidity, as these survivors often already had limited trust in the authorities. In these cases, it was described as pivotal to secure clearer roles, responsibilities and continuity among involved organizations.

“We had a complex case, where you needed to work partly based on the sexual trauma that she was exposed to, but also based on the self-harming behaviour. She was living at an institution … had contact with social services. Psychiatry then refers this girl to the health centre based on self-harming behaviour … without joint assessment. … In that case, who takes responsibility … the patient is thrown back and forth and … during waiting times … more situations occurred with risk for exposure to sexual trauma … before the actors in several organizations had decided what to do …”

– Participant, Health care services, Focus group 8

“Based on this case, with the need for trauma therapy … there are long referral waiting times and there we try to keep a contact until psychiatry can take over in that case. On some occasions, it may be possible to switch to a contact via the health centre, eventually, depending on the situation … but you try to keep a continuity until someone else can take over to build up a sense of security around it. But I would say it depends on the actual provider’s interest in the case.”

– Participant, Health care services, Focus group 8

A need for interlinked processes to establish trust among survivors was also described in other situations, for example in health care services. Participants from health care services described meetings with survivors during pregnancy who had extended needs of long-term support and often also trauma therapy to manage gynaecological examinations and vaginal birth. It was common that these women needed help to coordinate long-term support and care between different organizations.

Participants from the police organization stated that survivors often needed help with coordinating long-term support during and after the legal process. In these situations, survivors of sexual violence often had to seek help and navigate between different organizations themselves. Additionally, participants from health care and social services stated that survivors who had experienced sexual abuse online often had needs of long-term support, but that this often was not recognized during legal processes.

Participants from organizations that provided trauma therapy also stressed the need for more flexibility in the number of sessions provided based on individual needs as well as the need for more resources, access for children and adults in need of therapy, and long-term support after therapy has been completed. They described that it was common that survivors had to navigate to find peer-support solutions or private therapy outside the existing health care system by themselves.

### The potential of developing capacity, competence and teamwork in trauma care

Many participants from health care, social services and integrated services for children expressed a strong willingness to do more to provide support and care to survivors of sexual violence. At the same time, it was also common that these participants referred to the potential of teamwork and the importance of support from colleagues and time for their own recovery after challenging consultations.

They described challenges when listening to traumatic life stories about brutal sexual abuse and expressed strong needs of time for their own recovery. In addition, they described the potential and strength of working in teams in demanding consultations in cases of co-morbidity and when working with children. Participants working in health care services described limited teamwork, including support from colleagues and possibilities to learn from others in demanding consultations, especially those who provided long-term support or trauma therapy.

Participants in health care services and social services expressed frustration due to limited organizational capacity and their individual level of competence. They expressed limited knowledge regarding sexual violence and how to assess the need for support and care. For example, they expressed feeling frustration in meeting survivors with different kinds of trauma, including sexual violence, and that they did not have enough skills to provide trauma care.

“We see a great need in our own work to further deepen our knowledge of sexual violence and of the opportunities to provide trauma support.”

– Participant 35, Social services, electronic survey

“We experience that there is a lack of adapted reception/information/support for people with intellectual disabilities in all activities that take on victims of violence.”

– Participant 72, Health care services, electronic survey

Participants across the organizations stressed the need for an in-depth understanding concerning basic skills in trauma care, including how to recognize trauma symptoms and offer stabilizing treatment. Furthermore, participants working in health care, social services and integrated services for children stated there was a need for updated knowledge on evidence regarding effective treatment of PTSD.

Participants also emphasized the need for accessible information about other organizations’ capacity and competence in trauma therapy and medical treatment. They also described limited competence in identifying exposure to sexual violence and lack of knowledge of who to contact when trauma care is needed and when to do so.

“Many say that they provide trauma therapy, but are not trained in sexual trauma … The knowledge of sexual trauma is often poor among many therapists.”

– Participant 16, Health care services, electronic survey

## Discussion

This study contributes with results on how different organizations providing support and care for survivors of sexual violence call for a more integrated way of working to improve services on equal terms.

Our results highlight the need to apply existing systematic approaches to identifying exposure to violence. Participants in this study who represented social services and school health services reported that they seldom used systematic approaches for identifying sexual violence, even though they reported that they often encountered risk groups. Previous studies show that tools like self-reported questionnaires, such as SEXIT [33], can assist in addressing exposure to violence [15,34,35]. By helping survivors disclose experiences of sexual violence as early as possible, repeated exposure to sexual violence could be prevented and people’s health could ultimately be improved [2,11]. However, emphasizing existing risk assessment tools is probably not enough, since previous studies show that many factors affect providers’ ability to identify exposure to sexual violence. For example, health care professionals can be reluctant to inquire due to lack of time [16], feelings of discomfort or limited skills concerning the subject of abuse [16,36]. Our findings complement these issues since solutions for identifying exposure to violence also are needed at an organizational level, especially since many survivors need to be asked several times before they share experiences [11,15]. However, implementing routine screening in a multiorganizational setting where specialised care is not available is only valuable if an appropriate response can be mobilised. Routine screening needs further considerations since potential harms of screening may occur including fear of not being believed, or offered unhelpful or even harmful interventions [37,38].

Our findings show that seamless pathways including coordination of emergency social care support and long-term care existed in services for children with mental health problems. They referred to “one way into the system”, where all support and care was available and coordinated from the same platform. This integrated care model for children could guide development of future models for survivors exposed to sexual violence regardless of age and gender. However, future development needs to consider how to create pathways from screening to response in a rural and multiorganizational setting with many actors across institutions and disciplines.

The results of our study highlight the importance of organizing and coordinating existing supportive resources and establishing interlinked processes for trauma therapy and long-term support in a platform to build trust and improve access to care among survivors also confirmed in previous research [11]. For example, many participants in our study wished for an interlinked process for coordinating long-term support and access to trauma therapy within and between organizations to provide health and welfare services on equal terms regardless of legal processes, age or gender. A previous Swedish study reported that many survivors face systemic obstacles in receiving access to long-term support and trauma therapy [11]. Overall, this also reflects the need to continue developing models for integrating existing resources of support in and between different public organizations and interest groups more effectively, since many health care and social care systems also are overburdened and suffer from resource constraints.

The aforementioned systemic obstacles to providing long-term support and access to trauma therapy also bring to mind the potential of developing capacity, competence and teamwork in trauma care to improve health among survivors of sexual violence. Participants in our study stressed the need for an in-depth understanding in the subject of sexual abuse, trauma and therapy. Furthermore, they also highlighted the value of teamwork, recovering, and learning from others, as also reported elsewhere [11,19,39]. Taken together, this suggests that limited capacity and competence in trauma-informed care and trauma therapy is probably a national problem.

The results of the study include perspectives from eight organizations such as health care services, social services, school and student health services, the police, integrated services for children, private care and NGOs. Most previous studies regarding care and support in this field focus on single organizations [16,17,18], even though an integrated care model [40] in a multiorganizational setting is likely to be needed to solve this problem.

This study confirms the potential of the methodological approach of co-design when applying the principles of social science and a knowledge-based integrated care model in a multiorganizational setting to improve long-term support and trauma care for survivors of sexual violence. The social science perspective on the integrated care model describes integration of care as a coherent set of methods and models focusing on service delivery designed to create connectivity, alignment and collaboration within and between organizations [40]. In addition, the value of the solutions proposed through a co-design process, and of the experiences of those participating in that process, are argued to be better aligned if all actors who are intended to benefit from a service are part of the design work. Co-production is considered to strengthen the relationship between citizens and actors in services [41]. A previous study exploring trauma-informed co-production in the context of sexual violence in a single [primary care] organization stresses the need for close partnerships in work, flexibility, and transparency regarding power dynamics, especially since sharing experiences can re-trigger trauma [42].

Future research should explore how co-production approaches can be used when developing and evaluating integrated trauma-informed care and support services for survivors of sexual violence in a multiorganizational setting. In addition, future research should examine how to develop an interlinked process for coordinating long-term support and access to trauma therapy within and between organizations to support health and welfare services on equal terms regardless of legal processes, age or gender. Previous research has predominantly focused on acute care immediately after the violence has occurred [42], focusing on women [42,43] and on violence in intimate relationships or domestic areas [22]; it is thus important to include all genders and all kinds of relationships in future research.

## Methodological considerations

A strength of this study is that the participants represented many different organizations that provide support and care for survivors of sexual violence, such as the health care services, social services, the local police authority, school/student health services and integrated services for children. Transferability and generalisability are central for trustworthiness in qualitative studies. We believe that the complex environment included in our study supports the transferability and generalisability of the findings to regions in rural settings where specialised care for survivors of sexual violence are not available.

Few men were included in the study and the majority of the participants represented health care services. This could be seen as a limitation, although it mirrors the probable gender proportion and the actors providing support and care for survivors of sexual violence in Sweden.

Regarding the involvement of survivors with lived experience, NGOs were engaged to represent people with lived experiences of sexual violence, rather than engaging survivors during the co-design process. Representatives from NGOs thus represented survivors of sexual violence and contributed to the design of the study together with the interprofessional research team, strengthening the sensitivity and relevance of the questions and the overall trustworthiness. Qualitative and quantitative data from different sources enabled triangulation, strengthening both the construct validity and the internal validity of the study [28,44]. Reliability was considered by presenting the approach in the methods section, describing the steps in the analysis and linking the results to the transcripts and responses by presenting quotes [30].

## Conclusion

Eight organizations providing support and care call for the need to improve health and welfare services for survivors of sexual violence. It is essential to develop and apply systematic approaches to identifying exposure to sexual violence at both an individual and an organizational level. In addition, it is necessary to integrate existing resources and develop interlinked processes for trauma therapy and long-term support to improve access and establish trust for survivors of sexual violence. To be able to improve support and care services for survivors of sexual violence, it is vital to strengthen capacity, competence and teamwork in trauma care.

## Additional File

The additional file for this article can be found as follows:

10.5334/ijic.8995.s1 Supplementary file. Supplementary 1 and 2.
