# Supplementary material for: Potentials for Improving Support and Care to Survivors of Sexual Violence – a Case Study Within a Multiorganizational Setting in Sweden
Source: Int J Integr Care. 2025 Sep 9;25(3):26. doi: 10.5334/ijic.8995 (PMC12427615; doi:10.5334/ijic.8995)
Supplement: Supplementary file. — Supplementary 1 and 2. [file ijic-25-3-8995-s1.pdf]

Supplementary 1. Interview/topic guide for interviews and focus group discussions

When do you come into contact with people who need help, support and care when exposed to sexual violence?

- What does such a situation look like?
- What do you do?
- What are others doing in your organization?

What do you and your organization do when there is an *urgent* need for help, support or care?

What do you and your organization do when there is a need for *long-term* help, support or care? (e.g. sleeping difficulties, pain, anxiety, PTSD)

How are victims identified?

- By you and your organization?
- How could more victims be identified?

How do you and your organization collaborate with other units and organizations on support efforts? (e.g. need for care or treatment related to trauma symptoms after sexual violence)

Talk about trust (in those who provide support)

- What role does trust play in care? (exemplify)
- How can trust be improved, both towards your own organization and other actors?

Talk about accessibility (regarding help, support and treatment)

- What role does accessibility play in care? (exemplify)
- How can accessibility be improved, both in your own organization and with other actors?

Have you or your organization involved any of those who have been subjected to sexual violence to improve care, i.e. worked with user collaboration/patient involvement/peer support?

- How?
- Could it be of value, do you think?

Talk about resource use (and indirect collaboration)

- How can the use of resources regarding individuals exposed to sexual violence be improved, both within your own organization and in relation to other actors?

Final question
Is there anything else that you would like to add/supplement/clarify in the focus group discussion?

Supplementary 2. Questions in the electronic survey

1. Which workplace/unit do you belong to?
2. Which municipality or part of the county is the catchment area for your activities?
3. Which target groups is your activity aimed at? (alternatives: age, gender)
4. Can your organization offer support/treatment/care for those who have been subjected to sexual violence? (alternatives: yes, no, don't know)

- If yes to the previous question – what kind of support/treatment/care can you offer?
- If yes to the question above – at what stage can you offer support/treatment/care? (Emergency: e.g. crisis support, protection, securing traces, reporting to the police. Long-term: e.g. counselling, trauma assessment, trauma treatment.)
- If yes to the question above – how long can they get support/treatment/care through you?
- If yes to the question above – which professionals in your organization work with support/treatment/care of the target group?

5. Do you see a need for any intervention that is currently lacking for the target group?
6. Is there anything else you would like to inform us about regarding your organization's opportunities to offer support/treatment/care after sexual violence?
